# Effect of Erythropoietin on Serum Brain-derived Biomarkers after Carbon Monoxide Poisoning in Rats

**Published:** 2012

**Authors:** Shabnam Shahsavand, Amir Hooshang Mohammadpour, Ramin Rezaee, Effat Behravan, Ramin Sakhtianchi,, Seyed Adel Moallem

**Affiliations:** 1 *Department of Pharmacodynamics and Toxicology, School of Pharmacy, Mashhad University of Medical Sciences, Mashhad, Iran*; 2 *Pharmaceutical Sciences Research Centre, Mashhad University of Medical Sciences, Mashhad, Iran*; 3 *Medical Toxicology Research Centre, Mashhad University of Medical Sciences, Mashhad, Iran*

**Keywords:** Carbon monoxide poisoning, Erythropoietin, Myelin basic protein, S100β, Rat

## Abstract

**Objective(s):**

Erythropoietin has been shown to exert neuroprotective effects in a variety of CNS injury models. Elevation of serum S100β, as a glial damage marker and myelin basic protein (MBP) has been reported to occur in acute carbon monoxide (CO) poisoning. The aim of this study was to evaluate the effect of erythropoietin (EPO) on serum S100β and MBP levels after CO poisoning in rats.

**Materials and Methods:**

Rats were poisoned with a mixture of 3000 PPM CO in air for 65 min. After exposure, half of the rats received 5000 u/kg EPO and the rest received normal saline. At 3, 6, 12, 24, 48, 72, 144, and 336 hr after exposure samples were taken. Additionally, EPO was administered at three lower doses (625, 1250 and 2500 u/kg). The serum S100β and MBP levels were measured using immunoenzymatic colorimetric assay. Hemoglobin level was alsomeasured.

**Results:**

Serum S100β levels in CO poisoned rats were significantly higher compared to the control group [6 hr (*P*< 0.01), 12 hr (*P*< 0. 001), 24 hr (*P*< 0.001), 48 hr (*P*< 0.008) and 72 hr (*P*< 0.008)]. EPO administration could significantly prevent serum S100β elevations after 12 hr (*P*< 0.008) and 24 hr (*P*< 0.008) of CO poisoning. Serum MBP levels in CO poisoned rats were not significantly increased in comparison with the control group (*P*> 0.05). EPO significantly increased the hemoglobin levels.

**Conclusion:**

EPO could partially prevent neuronal damage. More studies are required to elucidate other aspects of these effects.

## Introduction

Carbon monoxide (CO) poisoning is one of the most fatal poisonings in many countries (1). CO poisoning leads to various effects that range from cardiovascular and neurobehavioral abnormalities at lower levels to unconsciousness or death after acute exposure to higher concentrations. The symptoms and prognosis of acute CO poisoning correlate unreliably with the amount of measured carboxyhemoglobin (COHb) at the time of hospital admittance. Because of frequently overlooked diagnosis, measuring COHb in suspicious settings cannot be valuable (1).

S100β, a part of the large calcium-binding S100 protein family in astroglial cells, is a biomarker that is elevated in central nervous system (CNS) injuries. It can be measured in the serum or cerebrospinal fluid (CSF) by employing immunoassay methods. The amount of S100β increment has been found to be valuable in predicting final outcome after brain damages. Elevations of S100β over the convinced threshold levels could be reliable to prediction of brain damage or mortality (2). Astroglial cells are as sensitive as neurons to hypoxic stress. Therefore, S100β that is released from injured astroglial cells into peripheral blood could in some way be a sign of neuronal damage (3). A normal S100β level constantly predicts the absence of major CNS damage. Increased serum S100β levels are not essentially related to neuroglia damage but could also reveal the current failure of the blood brain barrier (4, 5). S100β can be a probable biomarker and prognostic parameter in CO-poisoned rats (6). There are some reports of S100β elevation in conscious CO-poisoned rats and after treatment with hyperbaric oxygen its level is decreased significantly (7). It is reported that evaluation of glial and neuronal proteins in peripheral blood is an important indicator of delayed outcome after severe traumatic brain injury with the existence of factors like age, Glasgow Coma Score, and CT scan findings (8). One case report indicates that S100β could be used as a biomarker of CNS necrosis after CO poisoning. This could be useful for clinical assessment, prognosis and management of CO poisoned patients (9). S100β level represents all S100 proteins, including at least one S100β monomer, i.e. the sum of the two dimmers S100A1β and S100β. Most published reports are on the basis of this ‘‘sum concentration’’ (10). Increased S100β and neuron-specific enclose concentrations are related to loss of consciousness in CO poisoning and they could be valuable markers in the evaluation of hypoxic brain damage that is induced by CO poisoning (9). 

Myelin basic protein (MBP) is the major protein constituent of myelin, the dielectric phospholipids layer that surrounds the axons of neurons. Injury to white matter causes the release of MBP into the CSF and serum, where it has been found to remain elevated for up to 2 weeks post injury. Interestingly, MBP can cause the blood-brain barrier opening, thereby facilitating its own entry (and possibly that of other CNS-derived biomarkers) into the blood (11). In this model, CO-mediated oxidative stress causes chemical alterations in MBP, which initiates an adaptive immunological response that leads to a functional deficit (12). Elevated MBP concentrations in the CSF may represent a predictive marker of delayed encephalopathy from CO poisoning, leading to a more appropriate triage of patients with CO poisoning (13). 

Erythropoietin (EPO) has an important role in neurogenesis, neuroprotection, and functions as a neurotrophic factor in the CNS. Therefore, EPO could be a good candidate for treatment of disorders associated with neuronal injury (14). EPO decreases inflammatory mediators (15, 16), and thus has been suggested for various clinical indications such as stroke, multiple sclerosis (MS), schizophrenia, retinopathy, Parkinson’s disease, epilepsy, brain trauma and spinal cord injury (17). 

All together, it seems that measuring these brain injury biomarkers could be valuable in prognosis of CO poisoning and also to monitor potential therapies (like EPO) in CO poisoning.

## Materials and Methods

***Experimental design***

Male Wistar rats (n= 105) with average body weight of 250±10 g were used. Rats were placed ina 16-L Plexiglas chamber, which was ventilated with amixture of 3000 PPM CO in air for 65 min (7). Immediately after CO exposure all conscious rats were randomly divided into sixteen groups. The first eight groups received 5000u/kg EPO and the rest received normal saline at the same injection time. At3, 6, 12, 24, 48, 72, 144, and 336 hours post exposure rats were killed by decapitation and blood samples were taken (one group from CO-poisoned rats and another group from CO-poisoned and EPO treated rats). For each sampling time there was a correspondent control group that received normal saline.

To evaluate whether lower EPO doses might be also effective in preventing the rise of serum S100β and MBP levels, twenty more rats were poisoned similarly and randomly divided into four groups. Three groups were treated with EPO at lower doses (625, 1250 and2500u/kg) and one group was the CO-poisoned group. There was a negative control group that received normal saline injection only. These rats were killed by decapitation 24 hr after CO exposure. For all groups, blood samples were taken, processed to serum by centrifuging at 3000 rpm for 20 min and then stored at -20 °C until analysis. The serum S100βlevels were measured using a commercial immune-enzymatic colorimetric assay kit (Diametra Co, Italy). MBP levels were measured using another commercial immune-enzymatic colorimetric assay kit (Cusabio Co, China). 

***Hemoglobin count***

The hemoglobin count was determined using an arterial blood analyzer for samples taken from the decapitated rats. Samples were taken from all the rats at 24 hr (day1) and 168 hr (day7) after CO poisoning.

***Statistical analysis***

Data are shown as mean±SD. Basal S100β levels and its levels after CO exposure were tested by one-way analysis of variance among groups. The differences among groups were analyzed by Tukey-Kramer correction method. *P*< 0.05 was considered to be significant. Analyses were performed using the Statistical Package for Social Science 11.5 for Windows (SPSS).

## Results

***Effect of EPO on S100β elevation time profile after CO poisoning***

**Figure 1:**
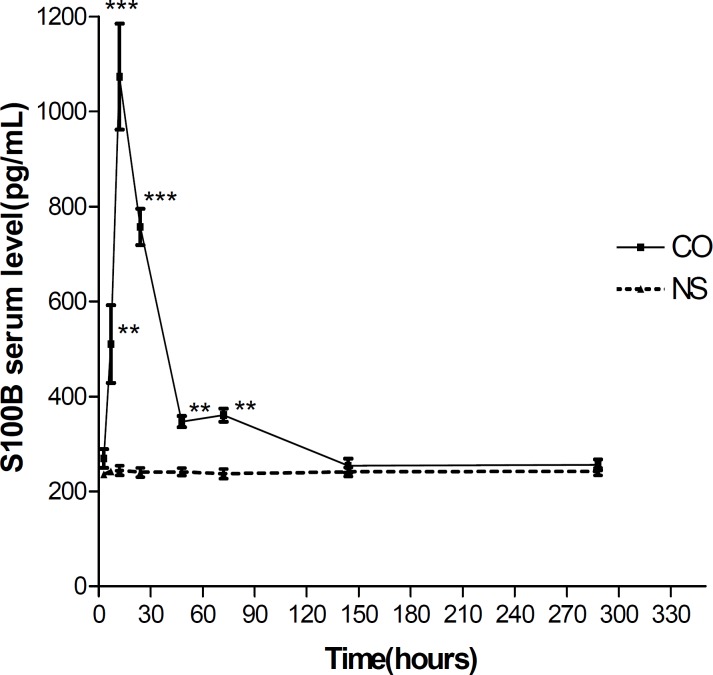
Mean serum S100β level at 3, 6, 12, 24, 48, 72, 144 and 336 hr after CO poisoning. * refers to comparison of serum S100β level in CO poisoned rats with normal saline injected ones at each time point (** and *** depict *P*< 0.01 and *P*< 0.001, respectively). Values are mean±SEM (n= 5).

Serum S100β levels in CO poisoned rats were significantly higher in comparison to the control group at 6 hr (212.5%, *P*< 0.01), 12 hr (447.22%, *P*< 0.001), 24 hr (315.41%, *P*< 0.001), 48 hr (144.58%, *P*< 0.008) and 72 hr (150.41%, *P*< 0.008) after CO poisoning. The elevation time profile is shown in Figure 1.

**Figure 2:**
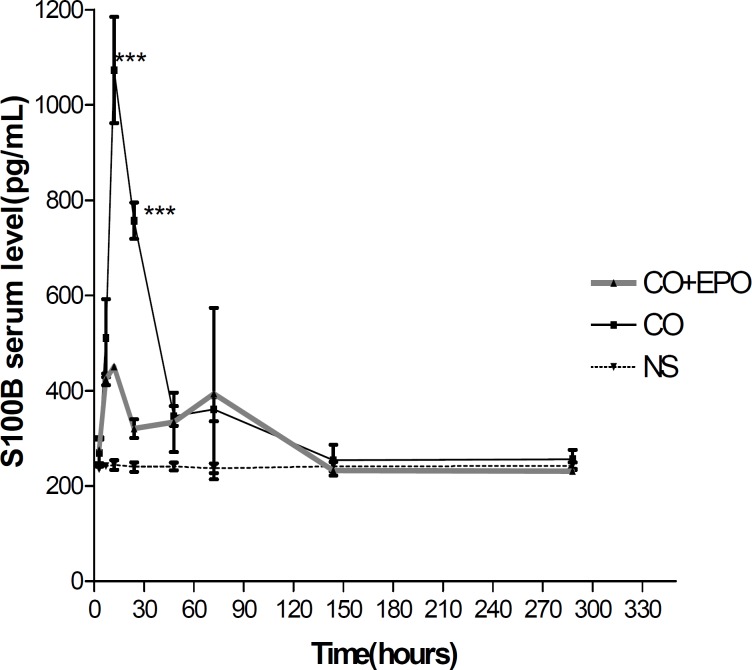
Mean serum S100β level at 3, 6, 12, 24, 48, 72, 144 and 336 hr after CO poisoning. * refers to comparison of serum S100β level in CO poisoned rats with EPO (5000u/kg) injected ones at each time point (*** depicts *P* <0.001). Values are mean±SEM (n=5).

EPO administration could significantly prevent the serum S100β elevation levels 12 hr (49.58%, *P*< 0.001) and 24 hr (50.6%, *P*< 0.001) after CO poisoning in comparison with the serum S100β levels in time matched CO poisoned groups, respectively (Figure 2). 

***Effect of different doses of EPO on S100*** **β** ***levels after 24 hr of CO exposure***

**Figure 3:**
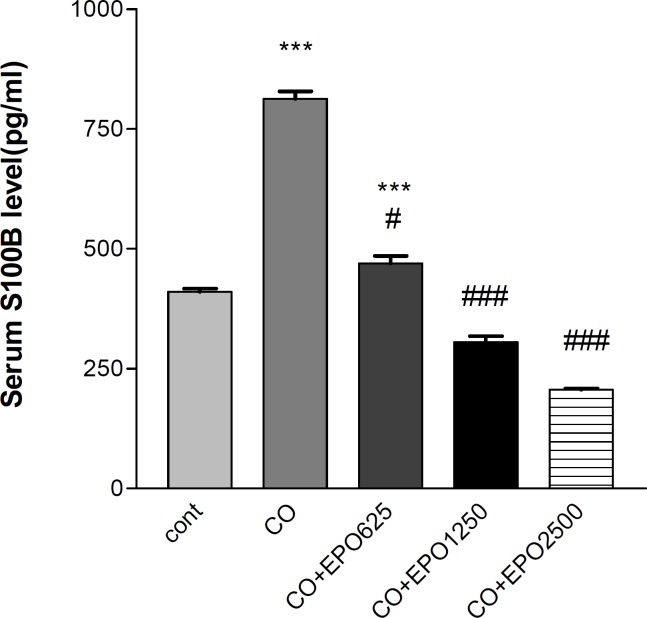
. Mean serum S100β level at 24 hr after CO poisoning. * is related to comparison of serum S100β level in CO poisoned rats with the control group (*** depicts *P*< 0.001). # refers to comparison of serum S100β level in CO poisoned rats with EPO injected ones at different doses (# and ### depict *P*< 0.05 and *P*< 0.001, respectively). Values are mean±SEM (n= 5).

EPO administration could suppress the serum S100β elevation after CO poisoning in a dose dependent manner. There was a significant increase (198.19%) in serum S100β levels following CO poisoning as compared with control animals (813.11 vs. 410.25 pg/ml, *P*< 0.001). EPO administration with doses 625, 1250 and 2500 u/kg prevented serum S100β elevation after CO poisoning by 42.24% (*P*< 0.05), 62.47% (*P*< 0.001) and 74.64% (*P*< 0.001) percents, respectively (Figure 3). 

**Figure 4:**
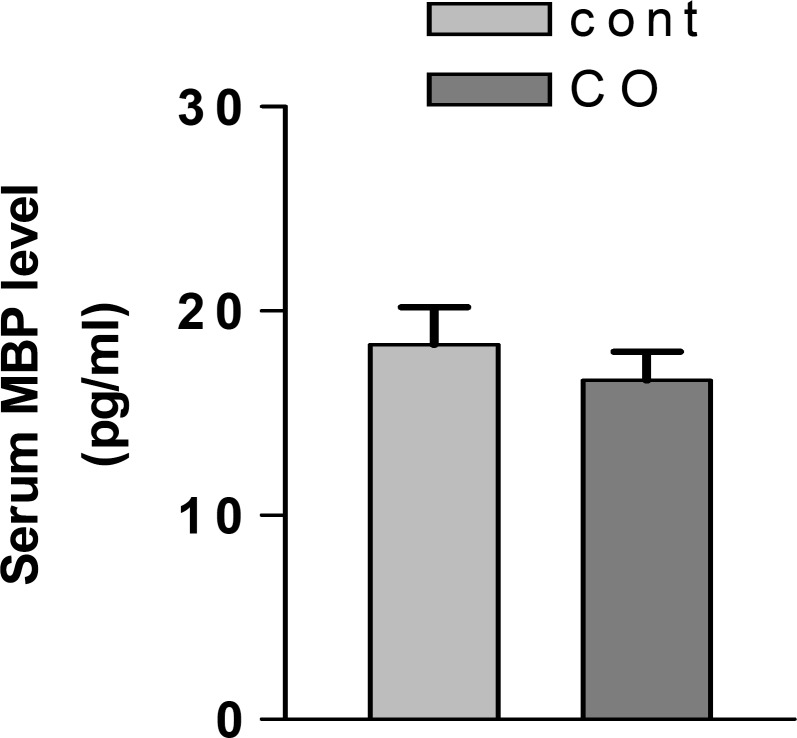
Mean serum MBP level at 24 hr after CO poisoning. The serum MBP level in CO poisoned rats (3000ppm for 60 min) was not significantly different in comparison to NS treated animals 24 hr after poisoning. Values are mean±SEM (n= 5). *P*> 0.05 as compared with NS treated animals (one-way ANOVA followed by Tukey–Kramer test).

**Figure 5:**
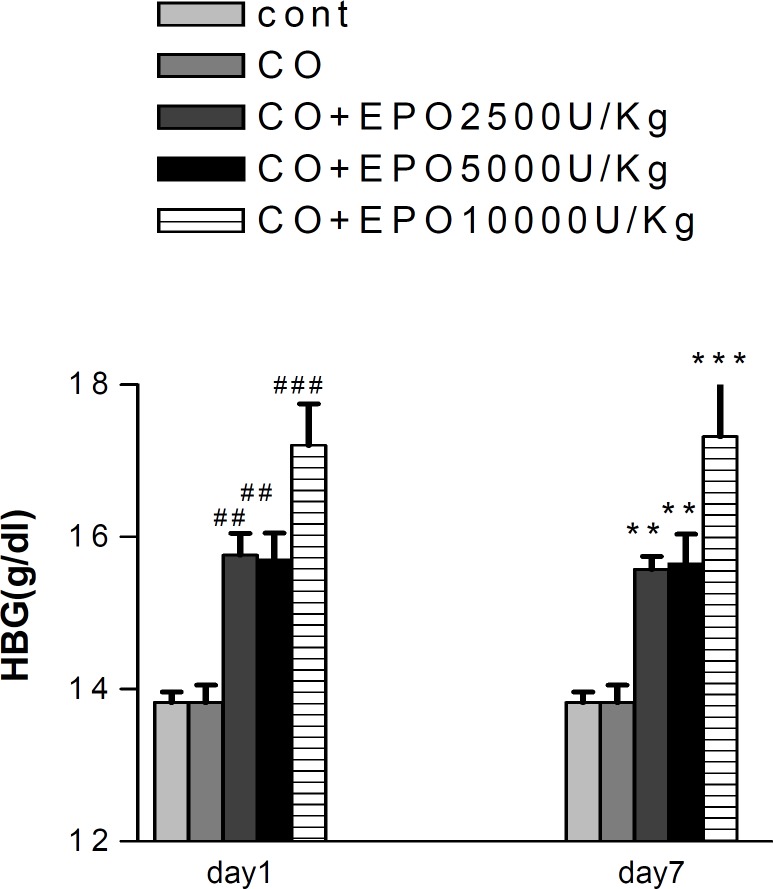
Effect of EPO on hemoglobin (g/dl) after CO exposure. ## (*P*< 0.001) and ### (*P*< 0.001) as compared with NS treated animals after 1day. ** (*P*< 0.01) and*** (*P*< 0.001) as compared with NS treated animals after 7days. (one-way ANOVA followed by Tukey–Kramer test). Values are mean±SEM (n= 5).

***Evaluation of MBP release into serum after CO poisoning***

All rats were killed by decapitation 24 hr after CO exposure. The mean serum MBP level in CO poisoned rats was not significantly increased in comparison with the control group (*P*> 0.05) (Figure 4). 

***Effect of EPO on hemoglobin (HBG) count***

EPO could significantly increase the hemoglobin count at 2500 and 5000 U/Kg (*P*< 0.01) and 10000 U/Kg (*P*< 0.001), at day 1 and 7 (168 hr) after administration in comparison to the control group. CO poisoning itself had no effect on hemoglobin count even after 1 day and 7 days (*P*> 0.05) (Figure 5).

## Discussion

The purpose of the present study was the evaluation of serum S100β elevation time profile after CO poisoning and the effect of EPO in this regard. The effect of different doses of EPO was also studied. The main findings were a significant rise in S100β serum levels after CO poisoning and a remarkable suppression of this elevation further to EPO administration. A similar temporal profile with an early maximum and a rapid decrease in serum S100β levels were seen in traumatic brain injured patients (10).

It is mentioned that EPO can directly reduce the risk of astrocyte swelling after stroke and other brain damages (18). Astroglial cells have water channel aquaporin 4 (AQP4). Glutamate, by affecting group I metabotropic glutamate receptors (mGluRs) can increase the permeability of astrocyte AQP4. Hypoxia-ischemia increases astrocyte water uptake. EPO antagonizes the effect of group I mGluR agonist on astrocyte water permeability. Activation of group I mGluRs triggers fast and highly regular intracellular calcium oscillations and EPO interferes with this signaling by varying the frequency of the oscillations. These effects are immediate, but other neuroprotective effects of EPO are dependent on gene activation (18). It is conceivable that EPO could prevent the elevation and then release of S100β from damaged astrocyte by this mechanism.

We found that there is another peak level of S100β after 72 hr of CO exposure. It is reported that S100β was significantly elevated in patients' cortexes with relapsing remitting MS (19). CO poisoning causes an autoimmune reaction against myelin. Myelin loss is a hallmark for MS. Investigators do mention that CO poisoning causes the same alterations in the basic constituent of myelin which is also seen in MS and these changes are considered to arouse the immune system to view the myelin as defective and thus attack it (20). Therefore, this second peak may be the consequence of autoimmune reaction against myelin. The S100β specificity for brain damage is compromised by its propensity to rise by injuries outside of the CNS. Soft tissue wounds, small fractures, and sprains cause minor increases in S100β with rapid normalization within 24 hr (17). More severe peripheral damages such as huge fractures and abdominal injuries significantly elevate the S100B levels. However, these elevations also have a short half life and a tendency to normalize after 24–48 hr. In contrast, moderate to severe brain damage has been related by considerable increments of S100β that last more than 48 hr. Thus, sustained increase of S100β beyond 48 hr might more precisely reflect the amount of brain damage with slight involvement of peripheral injuries (17). We found that the increment of S100β level lasts beyond 48 hr. Therefore, we might conclude that this elevation is the consequence of moderate to severe brain damage after CO poisoning. We took the blood samples for analyzing the dose dependency of the S100β elevation after 24 hr as this time point is not excessively early to cause false positive results and also at this time point its level is still significantly higher than the basal concentration. EPO could increase the hemoglobin level 24 and 168 hr after its administration. Therefore, the neuroprotective effects found from EPO in our evaluations could be a result of hemoglobin level increase and enhanced oxygen delivery to injured brain tissues. We assessed the MBP serum level after 24 hr to compare it with the serum S100β level. However, we found out that MBP serum level could not be a valuable marker in early diagnosis for brain injury after CO poisoning. 

When EPO is administered at doses needed for erythropoiesis (200–400 u/kg), it does not cross the intact blood brain barrier in detectable quantities (21). EPO in supra-pharmacological doses (2000–5000 u/kg per dose) can cross into the CNS of rats. Thus, we used 5000 u/kg of EPO for the first phase of our study. It is reported that systemic administration of high-dose EPO produces CSF concentrations of this drug ranging from 50 to 350 mu/ml at 3–3.5 hr in adult healthy rodents and nonhuman primates (21). The observation that EPO could significantly decrease S100β level at 12 hr after poisoning further support the above results. Our preliminary studies showed that even 2500 u/kg of EPO could after poisoning. Our preliminary studies showed that even 2500 u/kg could extremely decrease the S100β level after CO poisoning. Therefore, we found out that lower doses of EPO (625 and 1250 u/kg) also could cross into the brain due to the malfunctioning of the blood brain barrier in the case of poisoning and hypoxia. There is a case report of using EPO in the treatment of encephalopathy associated with CO poisoning (22). 

## Conclusion

We have shown that EPO could, at least partially prevent neuronal damage due to CO exposure. Thus, EPO might be a good candidate for treating serious and fatal poisonings with CO. However, it is necessary to study the effect of EPO on other aspects of CO toxicity and also the pharmacokinetics of systemically administered EPO under condition of CO poisoning should be also clarified. 
